# Graphene oxide immobilized 2-morpholinoethanamine as a versatile acid–base catalyst for synthesis of some heterocyclic compounds and molecular docking study

**DOI:** 10.1038/s41598-023-44521-9

**Published:** 2023-10-20

**Authors:** Leila Amiri-Zirtol, Tahereh Solymani Ahooie, Elham Riazimontazer, Mohammad Ali Amrollahi, Bibi-Fatemeh Mirjalili

**Affiliations:** 1 Department of Chemistry, Yazd University, Yazd, Iran; 2 Chemistry and Chemical Engineering Research Center of Iran, Tehran, Iran; 3 Biotechnology Research Center, Shiraz University of Medical Sciences, Shiraz, Iran

**Keywords:** Biocatalysis, Catalysis, Chemical safety, Green chemistry, Organic chemistry

## Abstract

In this study, a new heterogeneous catalyst was synthesized based on graphene oxide (GO) as a natural material. On the surface of nanosheet graphene oxide, 2-Morpholinoethanamine was immobilized using a non-toxic, green, and simple method. This resulted in the preparation of a bifunctional acid–base nanocatalyst. The synthesized composite was fully characterized using various methods, including Fourier transform infrared spectrometry (FT-IR), scanning electron microscopy (FESEM), energy dispersive X-ray spectroscopy (EDS), mapping, Raman spectroscopy, X-ray diffractometry (XRD), thermogravimetric analysis (TGA), and CHN elemental analysis. The catalytic reactivity of GO-mor was investigated in the one-pot synthesis of some benzo[*b*]pyran, pyrano[3,2-*c*]chromene, and polyhydroquinoline derivatives, yielding good efficiency and short reaction times. In addition, several recent studies have shown that some derivatives of pyran, chromene, and quinoline have remarkable anti COVID activity. Particularly, COVID-19 3CLpro/Mpro is considered a potential target for the treatment of this virus. For this purpose, docking models were constructed using the corresponding crystal structures with the synthesized derivatives. Based on the docking score and similarity of the binding mode to remdesivir and elvitegravir (the only approved drugs for the treatment of COVID-19), A_2_, B_1_, and C_4_ were selected as promising candidates for further research.

## Introduction

Organocatalyst research has drawn significant attention among scientists due to its innovative approach involving small organic molecules that enhance reaction conditions. To date, various substances, such as organic compounds, proteins, enzymes, botanical extracts, vitamins, and certain natural substances, have proven to be successful in serving as organocatalysts^1–4^. The key reason behind the development of innovative organocatalyst systems is their potential to align organic reactions with the principles of green chemistry. This is primarily because they do not rely on metals, unlike metallic catalysts. Organocatalysts offer several advantages, such as being cost-effective, non-toxic, and highly resistant to air and moisture. They are also easily obtainable and, in certain instances, affordable. Additionally, the necessity to remove the catalyst through washing is eliminated, as organocatalysts can be recycled, thus enhancing their environmental sustainability^5–7^.

Organocatalysts are categorized into a variety of small molecules, including pyridine, proline, and morpholine. Among these, 2-Morpholinoethanamine, a derivative of morpholine, stands out due to its remarkable nucleophilic potency at the nitrogen atom. This compound functions as a highly effective organocatalyst, exerting significant influence over a broad array of organic reactions, particularly those involving acylations^8–10^. For enhancing catalysts properties, they are fixed on different supports such as graphene oxide. Graphene oxide is considered to be highly effective as catalyst support because of its impressive characteristics such as a large specific surface area, lightweight nature, and the presence of diverse functional groups. Graphene oxide is also widely employed in applications like solar cells, sensors, nanosensors, and notably, as a catalyst. Its extended surface is rich in various functional groups, making it a valuable feature for customization and improvement.

To improve the catalysts, they are fixed on different supports. Graphene oxide represents a natural base that is considered one of the most promising candidates for a support due to its high specific surface area, low intrinsic weight, and various functional groups. Graphene oxide has been utilized in a wide range of applications, including solar cells, sensors, nanosensors, and notably, as catalysts. An essential characteristic of graphene oxide is its numerous functional groups present on its extensive surface, which plays a crucial role in its ability to undergo modifications^11–13^.

In 2020, Khazaee et al. used a (3-chloropropyl)-trimethoxysilane linkers to fix pipyrazine on the surface of graphene oxide, resulting in a novel base-acid catalyst named piperazine- GO. This catalyst was effectively used for the synthesis of pyrrole derivatives. However, the primary issue with this study was that creating the catalyst required multiple stages and the use of a harmful and costly toluene solvent, leading to an extended and labor-intensive manufacturing procedure^14^. Choudhury et al. developed a heterogeneous catalyst by applying (3-aminopropyl)-trimethoxysilane to the surface of graphene oxide to synthesize 1,4-dihydropyridines, acridinediones, and polyhydroquinolines. However, the use of expensive linkers and the preparation of the catalyst under unfavorable conditions, as well as the use of a hazardous solvent such as toluene, were clear limitations of this research^15^. On the other hand, there are multicomponent reactions (MCRs), which are of great importance due to the fact that the reaction can be carried out in one step, in a short time, with low cost and without the need for purification and separation of intermediate^16–18^.

A number of compounds, including pyrans, dihydropyranochromes, and polyhydroquinolines, can be synthesized by this method. Benzo[*b*]pyran, pyrano[3,2‑*c*]chromene derivatives exhibit biological activities such as antitumor, anticoagulant, antifungal, anticancer, antibacterial, and diuretic activities, and have medicinal properties in the treatment of Alzheimer's disease and amyotrophic lateral sclerosis^19–22^. Polyhydroquinolines are known in medicine for their diverse effects, which include antihypertensive, vasodilator, antimutagenic, antitumor, antispasmodic, antidiabetic, antianxiety, antidepressant, and analgesic effects^23,24^. Several approaches have been documented for the synthesis of these compounds. Various techniques, including microwaves, ultrasound, and the use of different catalysts like L-proline^25^, ZnO^26^, silica gel/ NaHSO_4_^27^, cyanuric chloride^28^, Yb(OTf)_3_^29^, γ-cyclodextrin (γ-CD)^30^, MNPs–PhSO_3_H^31^, SO_3_H@carbon^32^, ZIF@ZnTiO_3_^33^, bis(3-(piperazine-1-yl) propyl) tungstate^34^, Bismuth nitrate^35^, Silica-Bonded N-Propylpiperazine Sodium n-Propionate^36^, MCM-41 supported cobalt (II)^37^, Fe_3_O_4_@ MCM-41@ Cu-P2C^38^, can all be mentioned as potential methods^39–41^. However, these approaches have drawbacks, such as low efficiency, complicated catalyst separation, high cost of the catalyst and the use of hazardous chemicals in catalyst production, and long reaction times.

Considering the problems mentioned in this work, we aim to introduce a novel nanocatalyst utilizing graphene oxide as its foundation. This heterogeneous catalyst was created by anchoring the organic compound 2-aminomorpholine-ethanamine onto the graphene oxide nanosheet surface. The absence of harmful solvents, the ability to conduct the reaction under mild and eco-friendly conditions for stabilizing this organic substance, and the cost-effectiveness of this method are noteworthy and novelty of our research. In this approach, the composite was synthesized by leveraging the epoxy groups present on the surface of graphene oxide and incorporating an organic compound containing a nitrogen atom. The presence of free nitrogen and carboxylic acid groups in the graphene oxide wall created a heterogeneous acid–base nanocatalyst. The placement of 2-aminomorpholine on the surface of graphene oxide creates a gap between the graphene oxide layers. This gap allows easier access to the raw materials that react with the catalyst. By distributing 2-aminomorpholine on a large surface area such as GO, the performance of the synthesized catalyst is improved, which brings direct and substantial benefits. To determine its catalytic potential, the GO -mor composite was evaluated for its effectiveness in the synthesis of three different compounds with promising results. These results are indicative of the remarkable and satisfactory performance of the composite.

Respiratory tract infections (RTIs), which includes the recent severe acute respiratory syndrome coronavirus-2 (SARS-CoV-2), as the top global source of morbidity, mortality, economic disruption and public concern diagnosed by the World Health Organization (WHO) for research and development. With no specific treatment yet approved for COVID-19, it is valuable to explore existing drugs as potential therapeutics and to develop new agents with potential applications. Therefore, a priority for all scientists during this pandemic situation should be to ensure continuity to find novel clinical approaches against Covid-19. The availability of coronavirus macromolecule structures has prompted further efforts to identify possible anti-SARS-CoV-2 therapeutics through in silico analysis. Furthermore, to find out whether our synthesized compounds can cause inhibition of any target to combat COVID-19, though virtually only, we considered the main protease (M^pro^) or chymotrypsin-like protease (3CL^pro^) as a potential drug target in COVID-19, which is highly conservable among coronaviruses^42^. Main protease, also called 3CL^pro^ is one of the most prominent drug targets for the inhibition of CoV replication^43^. Therefore, the in silico molecular docking strategy was done to understand the binding mode of the synthesized ligands with the COVID-19 target protein 3CL^pro^/M^pro^^44, 45^.

## Experimental section

### Chemistry

All chemicals and graphite powder were procured from Fluka Chemical Co. (Switzerland) and Merck Chemical Co. (Germany) and utilized without any additional refinement. The reaction progress was assessed with the aid of thin layer chromatography (TLC) on silica gel PolyGram SILG/UV254 plates. Melting points were measured in open capillary tubes via a Buchi melting point B-540 B. The Shimadzu FT-IR 8300 spectrophotometer was deployed to characterize the synthesized compounds and catalysts. Furthermore, an XRD pattern was conducted using Ni-filtered X-rays with Cu-K radiation (λ = 1.54 Å) in the angular 2θ range of 4° to 70°. Additionally, the morphology of both GO and GO-mor was analyzed through field-emission scanning electron microscopy (FE-SEM) using MIRA3TESCAN-XMU. An energy-dispersive spectroscopy (EDS) analysis was also performed using SAMX MIRA II. Finally, the UniRAM Raman spectrometer equipped with a He-Cd gas laser (power 200 mW and excitation wavelength 325 nm) was utilized to record the Raman spectra. Elemental analysis was performed on a Heraeus CHN rapid analyzer.

### Synthesis and characterization

#### Synthesis of the graphene oxide- 2-morpholinoethanamine (GO-mor.)

**Figure 1:**
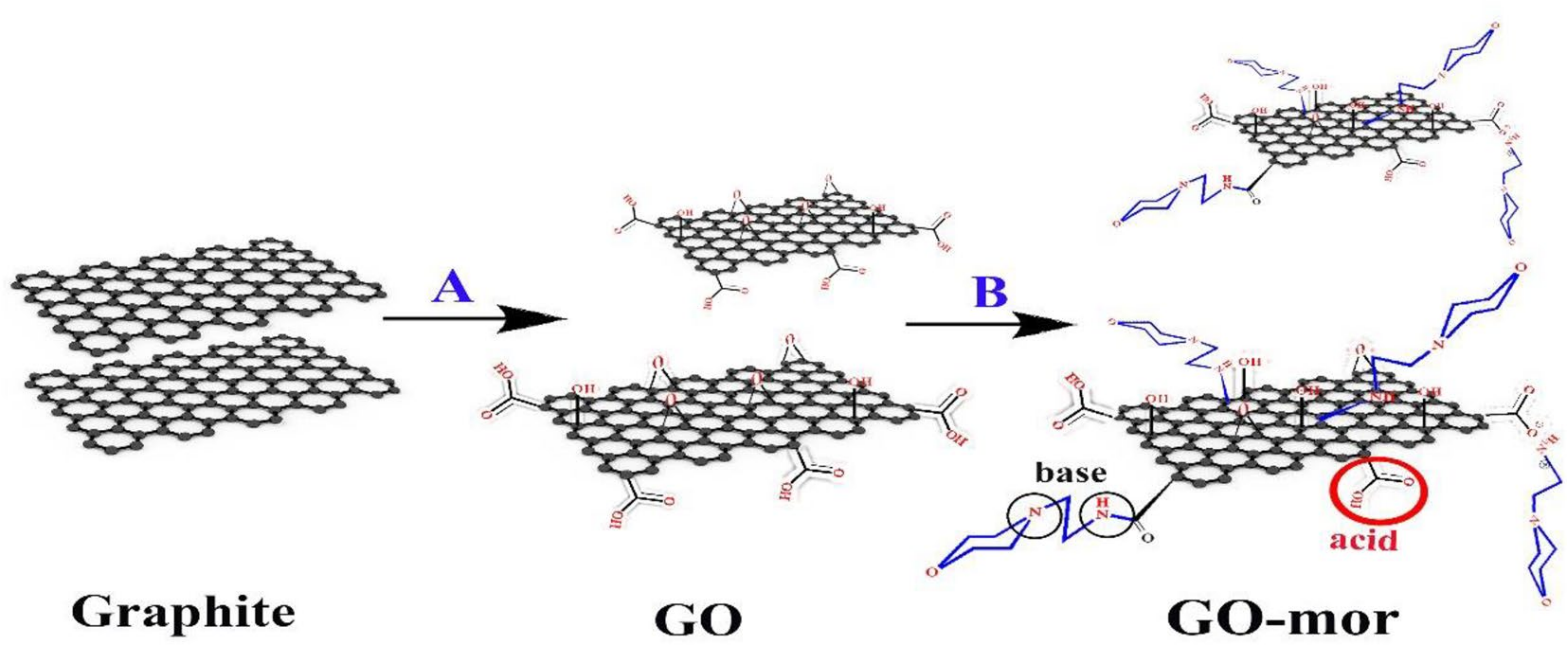
Preparation of Go-mor.

The The procedure began with the synthesis of graphene oxide by the Hammer method^46^. Subsequently, 0.5 g of graphene oxide was sterilized in ethanol and 1 ml of 2-morpholinoethanamine was added to the reaction vessel. The mixture was then refluxed for 24 h. After completion of the reaction, the catalyst was separated from the ethanol by centrifugation and then washed with hot ethanol. (Fig. 1).

#### General procedure for the synthesis of benzo[*b*]pyran derivatives

**Figure 2:**
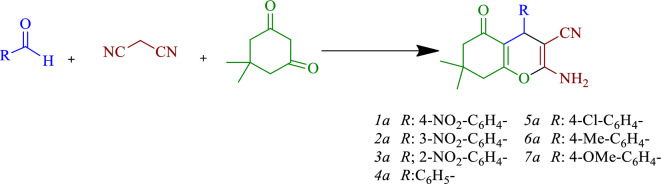
Synthesis of benzo[*b*]pyran derivatives.

In the experiment, arylaldehyde (1 mmol), malononitrile (0.066 g, 1 mmol), and dimedone (0.140 g, 1 mmol) were refluxed in a 50-mL balloon filled with EtOH/H_2_O solvent (1:1) along with 0.04 g of the catalyst. The progress of the reaction was monitored by TLC. After completion of the reaction, the catalyst was removed from the reaction solution by centrifugation. Upon cooling of the reaction solution, the pure product was precipitated and finally purified by recrystallization in ethanol (Fig. 2).

#### General procedure for the synthesis of pyrano[3,2‑*c*] chromene derivatives

**Figure 3:**
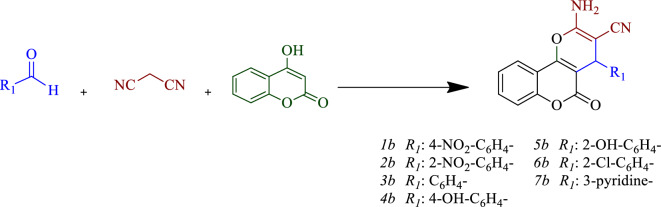
Synthesis of pyrano[3,2–*c*] chromene derivatives.

A solution containing arylaldehyde (1 mmol), malononitrile (0.066 g, 1 mmol), and 4-hydroxycoumarin (0.162 g, 1 mmol) was prepared in a 50-mL flask containing a solvent mixture of EtOH/H_2_O (1:1). The reaction mixture was then stirred with a catalyst (0.04 g). The progress of the reaction was monitored by TLC. After completion of the reaction, the catalyst was removed from the reaction solution by centrifugation. Upon cooling the reaction solution, the resulting pure product was precipitated and finally purified by recrystallization in ethanol (Fig. 3).

#### General procedure for the synthesis of polyhydroquinoline derivatives

**Figure 4:**
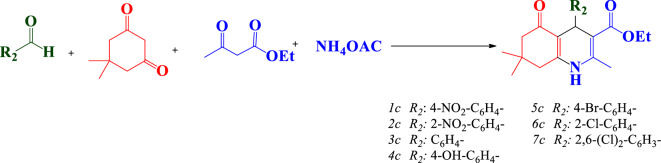
Synthesis of polyhydroquinoline derivatives.

At the beginning of the synthesis, arylaldehyde (1 mmol), dimedone (0.140 g, 1 mmol), ethyl acetoacetate (1 mmol), and ammonium acetate (1.2 mmol, 0.093 g) were refluxed in EtOH/H_2_O (1:1) together with the desired catalyst (0.04 g). The progress of the reaction was monitored by TLC. Once the reaction was complete, the solvent was removed and the product was dissolved in hot ethanol. The catalyst was removed by centrifugation, and the product was recrystallized from ethanol to give a pure sample (Fig. 4).

#### Docking simulation method

To select the best X-ray structure to use for docking simulation, validation docking was employed since various 3D-crystal structures for 3CLpro/Mpro are available in the Protein Data Bank (PDB) (http://www.rcsb.org). After searching the PDB for the 36 3D X-ray crystal structures of the COVID-19 3CLpro/Mpro, four files 5rf3, 5rgi, 6lze and 6m0k were chosen based on the root-mean-square deviation (RMSD) values as shown in Table 7. The ligand molecules were then sketched and energy was minimized, saved in pdbqt format, and both co-crystallized ligands and water molecules were removed from the protein. To finish the preparation for docking simulation, hydrogens were added to the protein and it was converted into the required pdbqt format by merging non-polar hydrogens with their corresponding carbons. Lastly, the Auto Dock Tools package (1.5.6) was used to plan the simulations.

A Linux operating system and bash scripting were used to run cross-docking simulations. Autodock Vina (1.1.2) was applied to dock within a 30 × 30 × 30 grid box centered on a co-crystallized ligand. The coordinates for 5rf3, 5rgi, 5lze, and 6m0k were as follows: [x = 6.62, y = −0.49, z = 18.28], [x = 6.50, y = −0.38, z = 18.40], [x = −11.08, y = 11.76, z = 69.36], and [x = −12.53, y = 11.13, z = 68.18], respectively. Exhaustiveness was set to 100, and the other docking parameters were set to their default values. At the end of the simulations, the best docking poses were chosen for further analysis of enzyme–inhibitor interactions.

## Results and discussion

Figure 1 illustrates the simple synthesis of the GO -mor catalyst, which involves several sequential steps. First, natural graphite powder was used to synthesize graphene oxide by the Hammer method^46^. Subsequently, this synthesized graphene oxide was modified with 2-morpholinoethanamine. By placing 2-morpholinoethanamine on the surface of nano-graphene oxide (GO -mor) without using toxic substances and solvents, a bifunctional acid–base catalyst is prepared. The positioning of the 2-morpholinoethanamine groups on the surface of the graphene oxide catalyst was confirmed by the IR -spectrum, which showed the opening of the epoxy ring.

The surface of graphene oxide was modified by covalent bonding with 2-morpholinoethanamine, which fixed an organic compound on the surface. This process created a gap between the graphene oxide layers. This not only improved the access to the 2-morpholinoethanamine molecule and increased the efficiency of the catalyst, but also improved the reusability of the catalyst by covalent bonding of 2-morpholinoethanamine groups. The large surface area of graphene oxide also contributed to the improved catalytic properties. The structure of the catalyst was analyzed and identified using various techniques such as IR, FESEM, EDS, mapping, Raman, XRD and TGA analysis. These results highlight the significant implications of this study.

**Figure 5:**
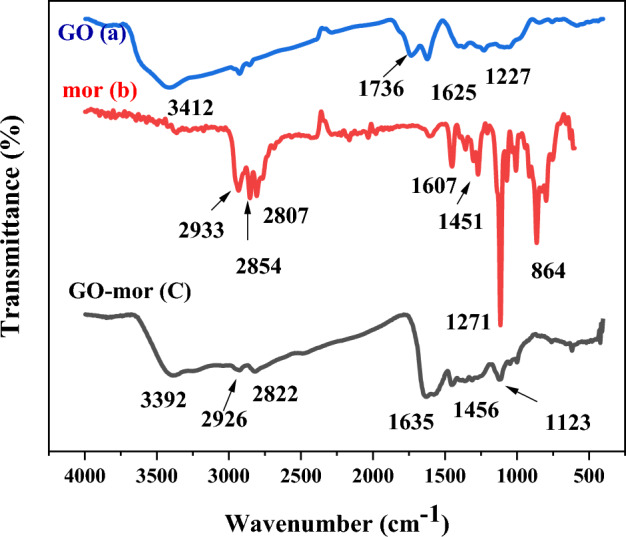
FT-IR spectra of GO (**a**), 2-morpholinoethanamine (**b**) and GO-mor (**c**).

The FT-IR spectra of graphene oxide, 2-morpholinoethanamine and functionalized graphene oxide with 2-morpholinoethanamine were depicted in Fig. 5. By comparing the FT-IR spectra of GO and GO-mor, it is possible to investigate the changes in the interaction between bands in graphene oxide before and after functionalization. In the graphene oxide's IR spectrum, the peak in the region 3412 cm^−1^ indicates the tensile vibrations of both the OH groups on the graphene oxide plate and the OH carboxylic acid groups of the GO wall. Additionally, two peaks are observed in the 1736 and 1625 cm^−1^ regions, representing the tensile vibrations of the C=O and C=C groups, respectively. In the region 1997 cm^−1^, peaks related to C–O epoxide, as well as C–O groups of alkoxide in the region 1227 cm^−1^, are also observed.

The FT-IR spectrum of 2-morpholinoethanamine displays various peaks, including peaks in the 2854 and 2807 cm^−1^ regions that correspond to CH_2_ groups^47^. The presence of 2-morpholinoethanamine on the graphene oxide surface is indicated by distinct changes in the spectrum, which reveal new peak formations and the removal of some peaks. Noticeable sharp peaks in the 1550, 1350, and 1150 cm^−1^ regions are also present. The peaks observed in 2822 and 2926 cm^−1^ belong to CH_2_, and their sharpness is apparent due to the presence of 2-morpholinoethanamine. Additionally, the interference of peaks between 2-morpholinoethanamine and graphene oxide in the 1000 cm^−1^ to 1561 cm^−1^ range is observed, causing changes in the spectrum of graphene oxide in this area.

**Figure 6:**
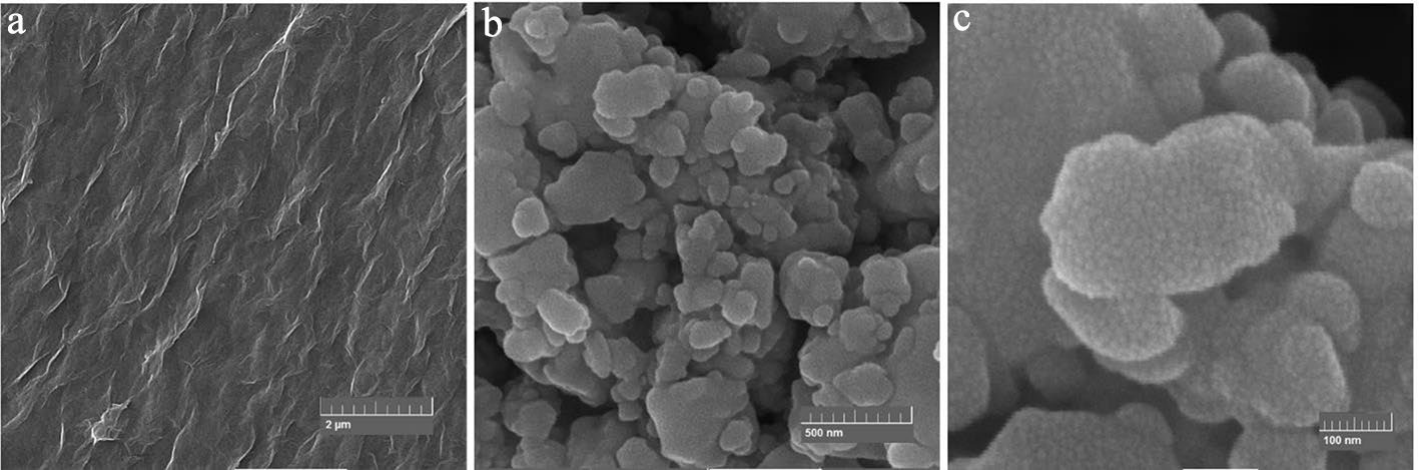
FESEM image of GO (**a**), GO-mor (**b,c**).

The morphology of GO-mor is presented in Fig. 6. The surface of the graphene oxide image shows noticeable irregularities and wrinkles that are attributed to oxygen groups residing on its surface (Fig. 6a). The FESEM data obtained from the functionalized graphene oxide surface revealed the presence of numerous spots, which confirmed the change in morphology due to the presence of 2-morpholinoethanamine particles on the surface (Fig. 6b, c).

**Figure 7:**
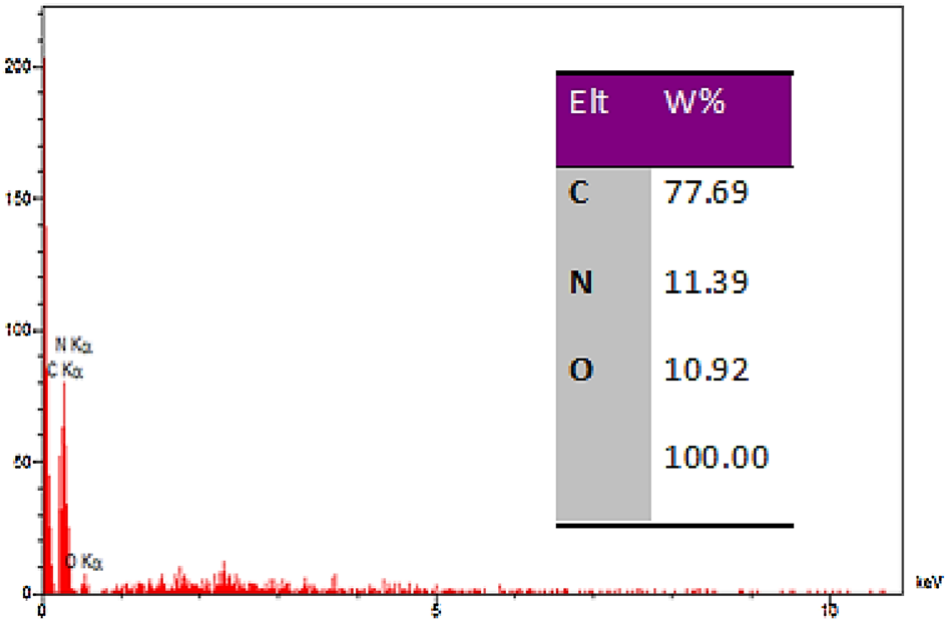
EDS analysis GO-mor.

The EDS analysis investigated and depicted in Fig. 7 reveals the presence of nitrogen within the structure of the catalyst, in addition to the oxygen, carbon, and hydrogen groups which are the main components of GO. This confirms the presence of 2-morpholinoethanamine in the catalyst. The percentages of C, N and O were detected as 77.69%, 11.39%, and 10.92%, respectively.

**Figure 8:**
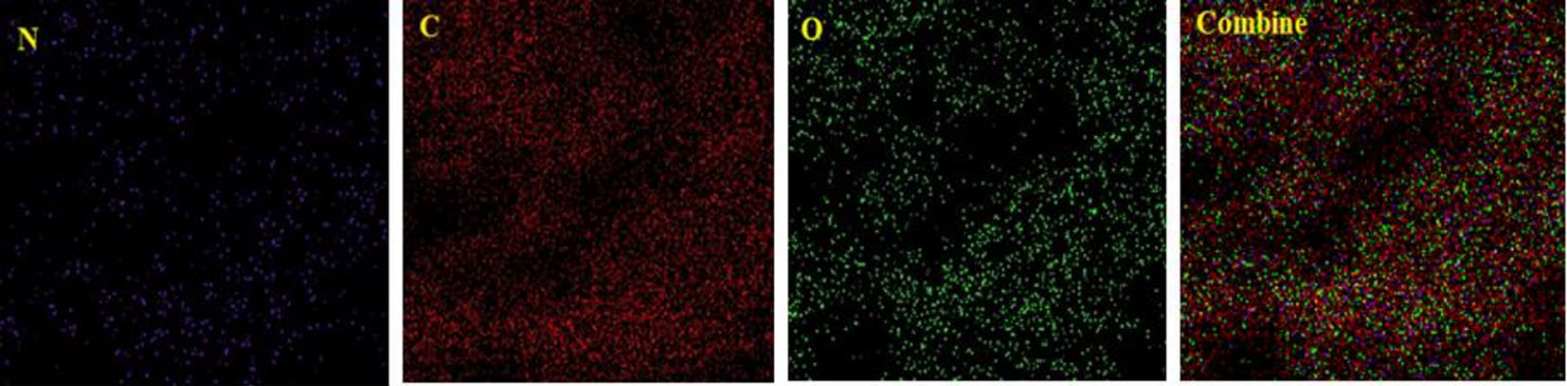
Elemental mapping images of GO-mor.

The elemental mapping images of the catalyst presented in Fig. 8 demonstrate a uniform distribution of the 2-morpholinoethanamine on the surface of graphene oxide.

**Figure 9:**
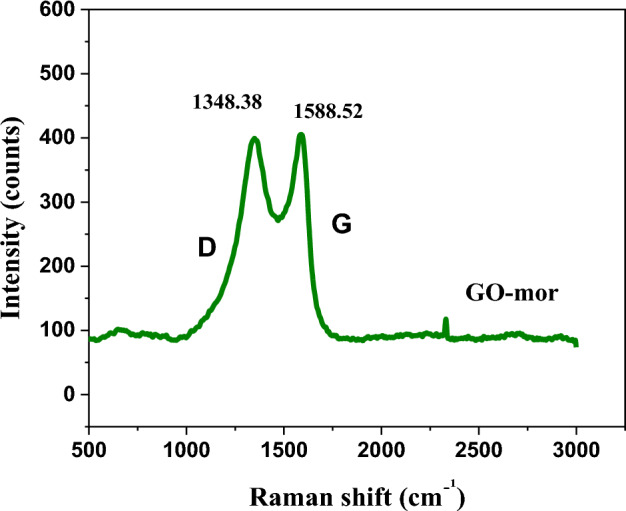
Raman spectroscopy GO-mor.

Given Raman test's capability to examine carbon structures effectively, the functionalized graphene oxide underwent Raman evaluation to discern its structural modifications as depicted in Fig. 9. Typically, the Raman spectrum of graphene oxide exhibits two clear-cut peaks, D and G that D correspons to sp^3^ carbon atoms while G corresponds to sp^2^ carbon atoms. Thus, to assess the sp^2^ size of a carbon structure possessing sp^3^ and sp^2^ bonds, evaluating the D and G band intensity ratio (ID/IG) can prove to be a valuable parameter. As depicted in Fig. 3b, in the functionalized graphene oxide, the G and D bands have shifted to 1588.52 and 1348.38 cm^-1^, respectively, with the D band being broader. Comparatively, the ID/IG ratio of functionalized graphene oxide (0.972) increased in contrast to that of GO (0.906) which indicates the entry of sp^3^ into the graphene oxide sheets and defects in its structure after functionalization^48^.

**Figure 10:**
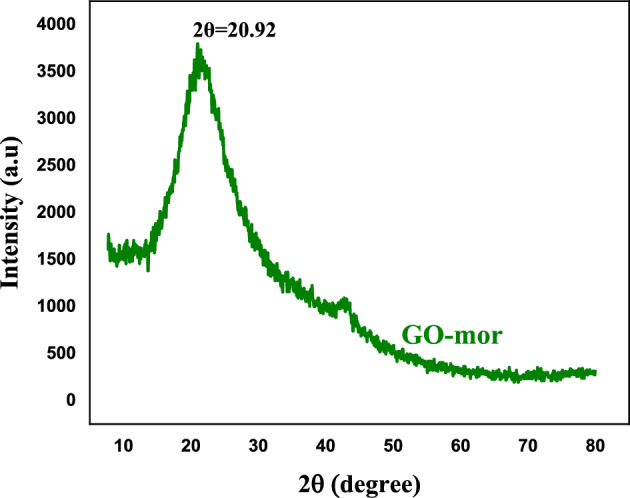
XRD pattern of GO-mor.

In Fig. 10, the XRD pattern of functionalized graphene oxide is presented, which enables the evaluation of the distance between GO-mor sheets. The analysis displays peak at 2θ = 20.9°, the inter-sheet distance of functionalized GO. A comparison with the XRD analysis of graphene oxide^11^ reveals an increase in distance between functionalized GO nanosheets (d = 1.75). The presence of functional groups, chain length, and ring in 2-aminomorpholine contribute to the observed increase in distance. Overall, the outcomes suggest that the introduction of organic molecules on the surface of graphene oxide nanosheets diminishes the interlayer interaction and elevates the distance between GO sheets. Such an increase in distance facilitates the access of reactants to the target molecule and enhances catalytic activity.

**Figure 11:**
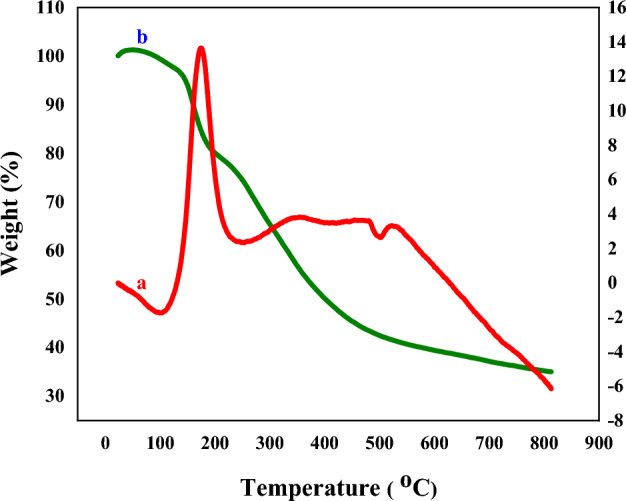
Thermal gravimetric analysis GO-mor pattern of (**a**) DTA, (**b**) TGA.

Figure 11 displays the TGA and DTA curves for 2-morpholinoethanamine functionalized grapheme oxide. Thermo-gravimetric analysis (TGA) indicates that weight loss occurs in three steps. The first weight loss (~ 100 °C) pertains to the moisture extraction from the catalyst structure. Mass loss commences at around 200 °C and subsists until about 483 °C, corresponding to the hydroxyl groups on the graphene oxide surface and the organic functional groups. This process indirectly reflects the quantity of 2-morpholinoethanamine on GO. Eventually, at higher temperatures, there is a mass loss that arises from the complete decomposition of grapheme oxide.

The elemental analysis of GO-mor was conducted, and the results of the CHN analysis revealed thet the weight percentages of carbon, nitrogen, and hydrogen were 33.32%, 4.33%, and 5.37% respectively. The presence of nitrogen confirms that the composite was synthesized have 2-morpholinoethanamine.

### Catalytic application

Following the preparation and characterization of the targeted catalyst, it was used for synthesizing benzo[*b*]pyran, pyrano[3,2-*c*] chromenes, and polyhydroquinoline derivatives. For this purpose, multiple experiments were conducted to determine the optimum conditions for synthesizing benzo[*b*]pyran and pyrano[3,2-*c*] chromenes derivatives. In this regard, the reaction of benzaldehyde, malononitrile, and dimedone chosen as the reaction model. We investigated the model reaction behavior, to evaluate the effect of 2-morpholinoethanamine. Initially, the reaction was studied in non-catalyst conditions, and then used graphene oxide, 2-morpholinoethanamine and GO-mor (0.04 g) as a catalyst. The differences between the reaction yields showed that GO-mor can be introduced as an effective heterogeneous catalyst for the synthesis of the model reaction (Table 1, Entries 1–4).

**Table 1:** Optimization the reaction conditions for synthesis of 1a.

Entry	Catalyst	G	Temp	Solvent	Time (min)	Yield (%)
1	–	–	50	EtOH	60	40
2	GO	0.04	50	EtOH	60	45
3	mor	0.04	50	EtOH	21	82
4	GO-mor	0.04	50	EtOH	12	96
5	**GO-mor**	**0.04**	**50**	**EtOH/H**_**2**_**O(1:1)**	**12**	**96**
6	GO-mor	0.04	50	H_2_O	30	90
7	GO-mor	0.04	50	Solvent-free	60	40
8	GO-mor	0.04	50	EtOH/H_2_O(1:1)	20	90
9	GO-mor	0.04	r.t	EtOH/H_2_O(1:1)	34	80
10	GO-mor	0.04	Reflux	EtOH/H_2_O(1:1)	12	96
11	GO-mor	0.03	50	EtOH/H_2_O(1:1)	12	90
12	GO-mor	0.02	50	EtOH/H_2_O(1:1)	38	86
13	GO-mor	0.01	50	EtOH/H_2_O(1:1)	60	75

Significant values are in bold.

After comparing the efficiency of these three catalysts concerning reaction rate and product yield, the effectiveness of 2-morpholinoethanamine in the synthesized catalyst was verified. Subsequently, the impact of solvent, which is a vital aspect of chemical reactions, was investigated.

The selection of a suitable solvent plays a crucial role in both the safety of the reaction and its economic efficiency. Consequently, in the second step, several solvents (e.g., water, ethanol, and solvent-free conditions) were tested to determine their impact on the reaction process. The optimal solvent was identified as H_2_O/EtOH for the reaction (Table 1, Entries 4–7). Furthermore, the reactions were investigated at various temperatures. As shown in Table 1, The reaction was carried out at three different temperatures: 50°C, room temperature, and reflux. Among these, the temperature of 50°C was selected for conducting the reaction.

Finally, to optimize the amount of catalyst necessary for promoting the reaction, multiple trials were executed with varying amounts of the catalyst (Table 1, Entries 11–13). After scrutinizing the efficacy of distinct weights of the targeted catalyst, it was concluded that 0.3 g of graphene oxide-2-morpholinoethanamine catalyst was best suited for the desired reaction. Consequently, the ideal conditions for promoting the reaction were realized via the use of EtOH /H_2_O as a solvent, at 50 °C temperature, and with the presence of 0.4 g of the desired catalyst (Entre 11).

The even distribution of graphene oxide, which produces a more spaced arrangement of 2-morpholinoethanamine-graphene oxide sheets, significantly enhances the catalytic activity for targeting organic compounds. Therefore, this is one of the crucial factors that influence the progression of the reaction process.

### Comparison of the catalytic activity

After optimizing the model reaction and identifying the best-suited conditions, the desired compound derivatives were successfully synthesized. Summary data pertaining to these derivatives have been tabulated in Tables 2 and 3. Upon evaluating the reaction outcomes of different derivatives, it has been observed that electron-withdrawing groups on the aldehyde augment the reaction rate or efficiency in comparison to the electron-donating groups. This is attributed to the aldehyde carbonyl group, which facilitates better interaction with the catalyst.

**Table 2:** Synthesis of benzo[*b*]pyrans in the presence of GO-mor.


Entry	R	Time (min)	Yield (%)^b^	m. p. (ref.)
1a	4-NO_2_-C_6_H_4_–	12	96	181–217^31^
2a	3-NO_2_-C_6_H_4_–	10	95	210–213^31^
3a	2-NO_2_-C_6_H_4_–	12	95	225–228^19^
4a	C_6_H_5_–	12	92	228–231^19^
5a	4-Cl-C_6_H_4_–	10	98	205–206^19^
6a	4-Me-C_6_H_4_–	16	95	218–220^19^
7a	4-OMe-C_6_H_4_–	16	90	201–205^19^

1 mmol of 1, 2 and 3 in EtOH/H_2_O. All yields are isolated products. Compound 1a was checked by HNMR and CHN analyze.

**Table 3:** Synthesis of pyrano[3,2–*c*] chromenes in the presence of GO-mor.


Entry	R1	Time (min)	Yield (%)^b^	m. p. (ref.)
1b	4-NO_2_-C_6_H_4_–	10	96	247–249^32^
2b	2-NO_2_-C_6_H_4_–	15	95	258–260^32^
3b	C_6_H_4_–	18	93	248–250^33^
4b	4-OH-C_6_H_4_–	12	92	271–273^34^
5b	2-OH-C_6_H_4_–	16	92	267–270^35^
6b	2-Cl-C_6_H_4_–	15	95	273–275^34^
7b	3-pyridiyl	12	96	250–252^36^

1 mmol of 1, 2 and 3 in EtOH/H_2_O. All yields are isolated products.

**Figure 12:**
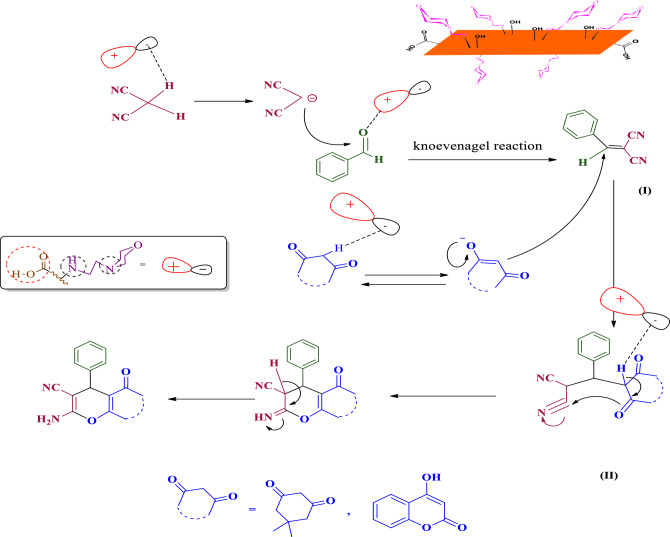
The proposed mechanism for GO-mor catalyzed one-pot synthesis of pyran derivatives.

Figure 12 illustrates the suggested reaction process for creating pyran derivatives with the aid of a bifunctional acid–base catalyst. Initially, Arylidene malononitrile (I) is produced via the Knoevenagel reaction. Subsequently, this preferred catalyst prompts the conversion of the enol/enolate form of β-dicarbonyl, engaging in a reaction with the alkene. In the final stage, the desired product is formed through an intramolecular cyclization.

In the following, to investigate the possible influence of the synthesized catalyst on the production of polyhydroquinoline derivatives, a reaction model was chosen Benzaldehyde. dimedone, ethyl acetoacetate, and ammonium acetate were employed in this reaction model. The reaction was initially performed under a variety of conditions (Table 4). The catalytic efficiency and duration of the reaction model were evaluated via three different reaction conditions: the absence of a catalyst, the utilization of graphene oxide as a catalyst, and the utilization of graphene oxide modified with 2-morpholinoethanamine as a catalyst (Table 4, Entries 1–4). The investigation of the reaction was carried out under three different solvent conditions, in the absence of a solvent, using ethanol as a solvent, and utilizing water as a solvent. The solvent mixture of EtOH/H_2_O (in a 1:1 ratio) was chosen as the preferred option due to its economic efficacy and the environmental sustainability of the reaction.

**Table 4:** Optimization the reaction conditions for synthesis of *1c*.

Entry	Catalyst	g	Temp	Solvent	Time (min)	Yield (%)
1	–	–	60	EtOH	60	40
2	GO	0.04	60	EtOH	45	65
3	Mor	0.04	60	EtOH	30	83
4	GO-mor	0.04	60	EtOH	12	93
5	GO-mor	0.04	60	EtOH/H_2_O (1:1)	20	93
6	GO-mor	0.04	60	Sovent-free	15	93
7	GO-mor	0.04	60	H_2_O	30	80
8	GO-mor	0.04	r.t	EtOH/H_2_O (1:1)	30	85
9	GO-mor	0.04	Reflux	EtOH/H_2_O (1:1)	12	98
10	**GO-mor**	**0.03**	**Reflux**	**EtOH/H**_**2**_**O (1:1)**	**12**	**98**
11	GO-mor	0.02	Reflux	EtOH/H_2_O (1:1)	18	83
12	GO-mor	0.01	Reflux	EtOH/H_2_O (1:1)	30	65

Significant values are in bold.

The quantity of the desired catalyst was explored by carrying out the reaction using four different quantities (0.04, 0.03, 0.02 and 0.01). The quantity of 0.03 g was deemed as appropriate for the reaction. Eventually, the optimal conditions for synthesizing polyhydroquinolines by means of the desired catalyst were determined to be at reflux temperature, employing the EtOH/H_2_O (in a 1:1 ratio) solvent mixture and utilizing a quantity of 0.03 g of the catalyst (Table 4, Entries 4–7).

Finally, the model reaction was performed in several different amounts of 0.01, 0.02, 0.03 and 0.04 g of GO-mor catalyst. The use of 0.03 g of the catalyst yielded optimal results in the reaction (Table 4, Entries 9–12). The best condition for the model reaction is EtOH/H_2_O solvent with 0.03g of catalyst at reflux temperature.

Under the optimal conditions, diverse derivatives of the compound were synthesized, and Table 5 was prepared to summarize the efficacy and the reaction time required for the synthesis of each compound. Notably, the incorporation of electron-withdrawing groups was observed to continue to enhance the reaction progress while minimizing the reaction time.

**Table 5:** One-pot four component synthesis of polyhydroquinolines under the optimized conditions.


Entry	R2	Time (min)	Yield (%)^b^	m. p. (ref.)
1c	4-NO_2_-C_6_H_4_–	12	98	181–217^37^
2c	2-NO_2_-C_6_H_4_–	10	98	191–194^37^
3c	C_6_H_4_–	15	93	199–200^38^
4c	4-OH-C_6_H_4_–	15	91	215–216^39^
5c	4-Br-C_6_H_4_–	13	97	213–215^40^
6c	2-Cl-C_6_H_4_–	12	95	216–218^37^
7c	2,6-(Cl)_2_-C_6_H_3_–	10	95	249–251^40^

1 mmol of 1, 2, 3 and 4 in EtOH/H_2_O. All yields are isolated products. Compounds 1c, 3c and 4c were checked by HNMR, CNMR and CHN analyze.

**Figure 13:**
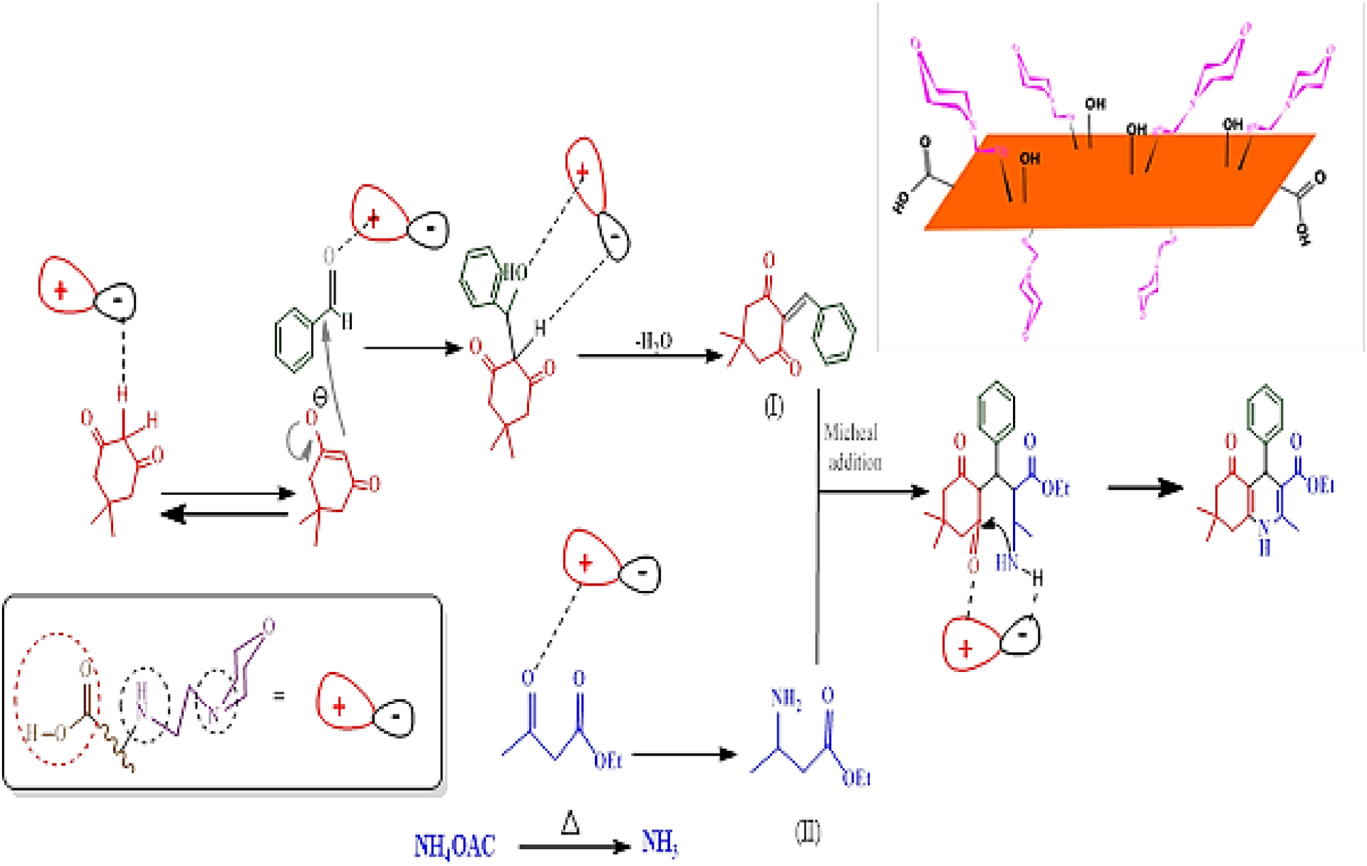
The proposed mechanism for GO-mor catalyzed one-pot synthesis of polyhydroquinoline.

The reaction proceeds in several steps. First, a new condensation takes place, followed by a Michael addition that produces intermediate 3. Finally, an intramolecular reaction occurs, leading to ring closure. Throughout these stages, the electron pair on nitrogen acts as a basic component, facilitating the removal of acidic hydrogens during the reaction and accelerating the reaction process (Fig. 13).

### Recycling and reusing the catalyst

The comparison between the efficiency of GO-mor and other catalysts reported for the synthesis of benzo[*b*]pyran, pyrano[3,2-*c*] chromene, and polyhydroquinoline derivatives in the present study and the research conducted in the previous years was presented in Table 6. Through this comparative analysis of our work with similar studies, it is readily apparent that the utilization of the desired catalyst in this reaction is highly effective, improving both reaction efficiency and reaction rate with gentle and appropriate working conditions.

**Table 6:** Comparison of the efficiency of GO-mor and other catalysts reported for the synthesis of benzo[*b*]pyrans and pyrano[3,2-*c*] chromenes and polyhydroquinoline.

Entry	Catalyst/temp/time (min)/solvent	Yield(%)	Ref

**1**	Zn^2+^/4A/reflux/4h/EtOH	98	^41^
**2**	Fe_3_O_4_@l-proline/reflux/15/H_2_O	44	^42^
**3**	MnFe_2_O_4_@SiO_2_NHPhNH_2_-PTA/80°C/30/solvent-free	95	^43^
**4**	Titania-supported sulfonic acid (n-TSA)/80 °C/45/solvent-free	96	^44^
**5**	GO-mor./50 °C/12 min/ H_2_O/EtOH(1:1)	96	This work


**1**	*o*-Benzenedisulfonimide (OBS)/120 °C/50/solvent-free	85	^45^
**2**	Zn_3_(PO_4_)_2_⋅4H_2_O/reflux/15/EtOH	89	^46^
**3**	PS-PTSA/80 °C/2h/EtOH	94	^47^
**4**	P_4_VPy/70 °C/5/ EtOH:H_2_O(1:1)	96	^48^
**5**	GO-mor./50 °C/10 min/H_2_O/EtOH(1:1)	96	This work


**1**	Ru^III^@CMC/Fe_3_O_4_/80 °C/20/solvent-free	92	^38^
**2**	SBA-15@AMPD-Co/100 °C/35/solvent-free	97	^40^
**3**	Fe_3_O_4_@SiO_2_-PEG/NH_2_/100 °C/10/solvent-free	99	^49^
**4**	PMO-ICS-PrSO_3_H/reflux/20/EtOH	95	^50^
**5**	GO-M./reflux/12 min/EtOH/H_2_O (1:1)	98	This work

**Figure 14:**
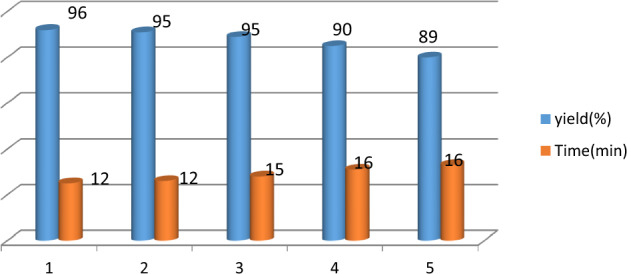
Catalyst recycling experiment of GO-mor in synthesis of 1a.

The recoverability and reusability of the catalyst, which is very important in green processes, was evaluated for the model reaction in pyrano[3,2–*c*] chromene derivatives (Fig. 14). The catalyst recovered from the model reaction, washed in hot ethanol and dried at 60 °C overnight and then the reaction was performed 5 times under the same conditions. It was observed that the activity of the catalyst did not decrease significantly after 5 periods.

**Figure 15:**
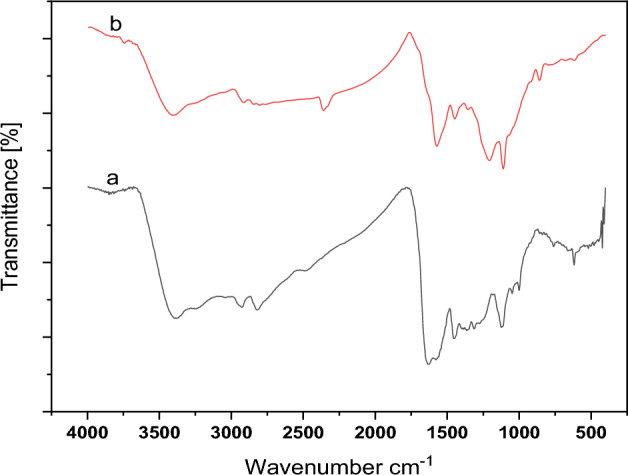
FT-IR spectrum of GO-mor before (**a**) and after (**b**) reuse.

As expected, little change in catalytic properties occurred, and it can be reused multiple times. Due to the formation of a covalent bond between 2-morpholinoethanamine and the graphene oxide surface prohibits the separation of the organic compound. Figure 15 offers a comparison between the initial FT-IR catalyst and the washed catalyst.

### Docking study with COVID-19 3CL^pro^/M^pro^

In order to explore potential treatments for COVID-19, 36 PDB codes of COVID-19 3CL^pro^/M^pro^ from the Protein Data Bank were selected as potential target receptors for docking screening. It could be considered worthy to predict if the ligands have a binding mode to the main protease (M^pro^) or chymotrypsin-like protease (3CLpro) as a potential drug target and act as COVID-19 M^pro^ inhibitors. To perform cross-docking of the compounds of interest in this study, 4 PDB codes were extracted from the Protein Data Bank. These proteins have heterocycles ligands comparatively like our synthesized structures and also the PDB IDs have been used in the previous studies related to COVID-19^51–57^. Four PDB codes (5RF3, 5RGI, 6LZE, and 6M0K) were chosen based on the criteria of RMSD < 2 (Fig. 7). Cross-docking with newly synthesized molecules was then investigated to identify the best energy. The 5rgi and 6lze PDB codes proved to be the best option for remdesivir and elvitegravir, the effective treatment for severe acute respiratory syndrome coronavirus 2 (SARS-CoV-2) infection, due to its highest score in binding energy. Thus, it can be used as an analysis model of interaction study.

**Figure 16:**
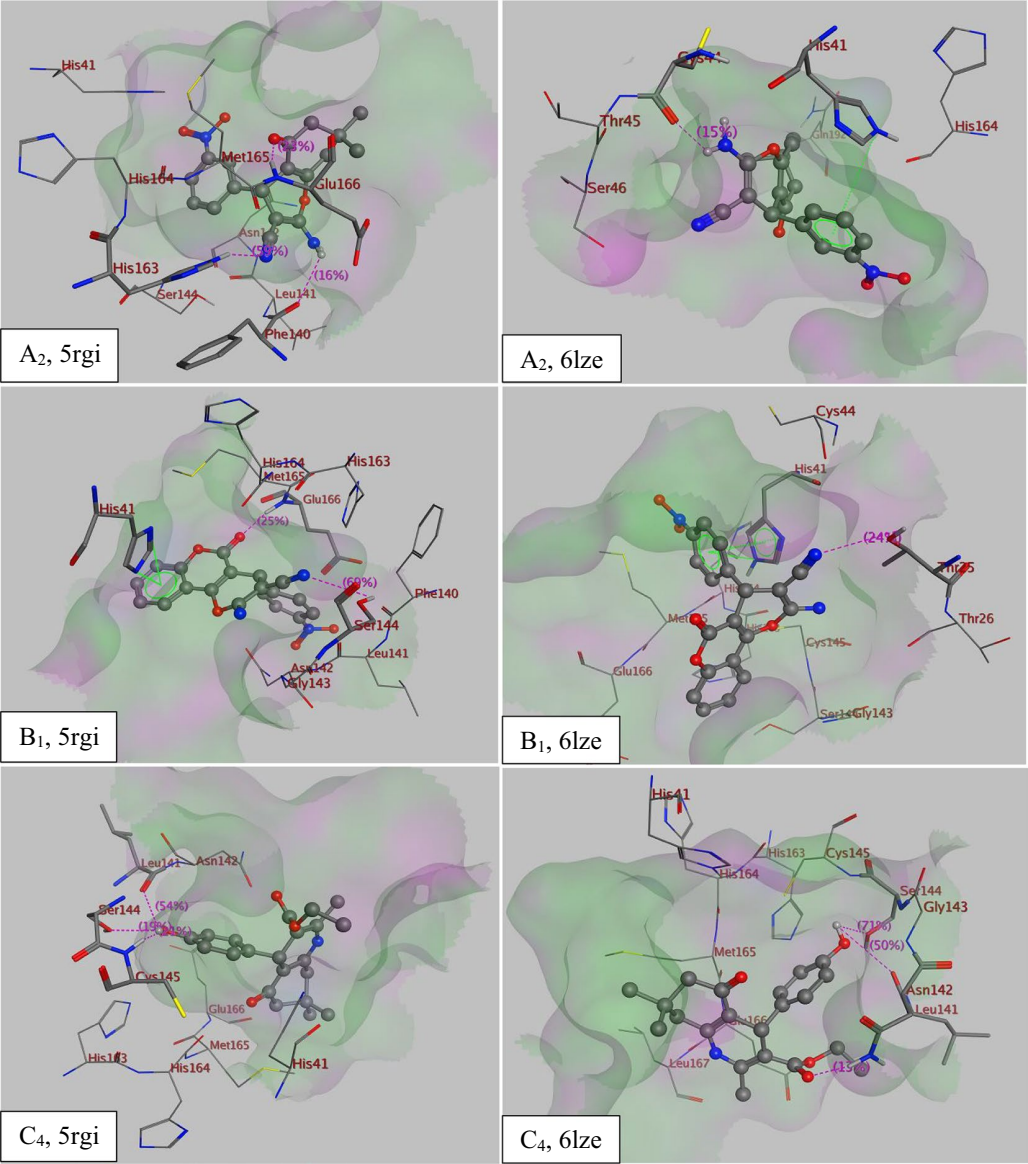
The best pose of the most active compounds A_2_, B_1_ and C_4_ with different X-ray crystal extructures (PDB code: 5rgi and 6lze) in the active site of COVID-19 3CL^pro^/M^pro^; and the residues of the active site involved in ligand binding.

**Figure 17:**
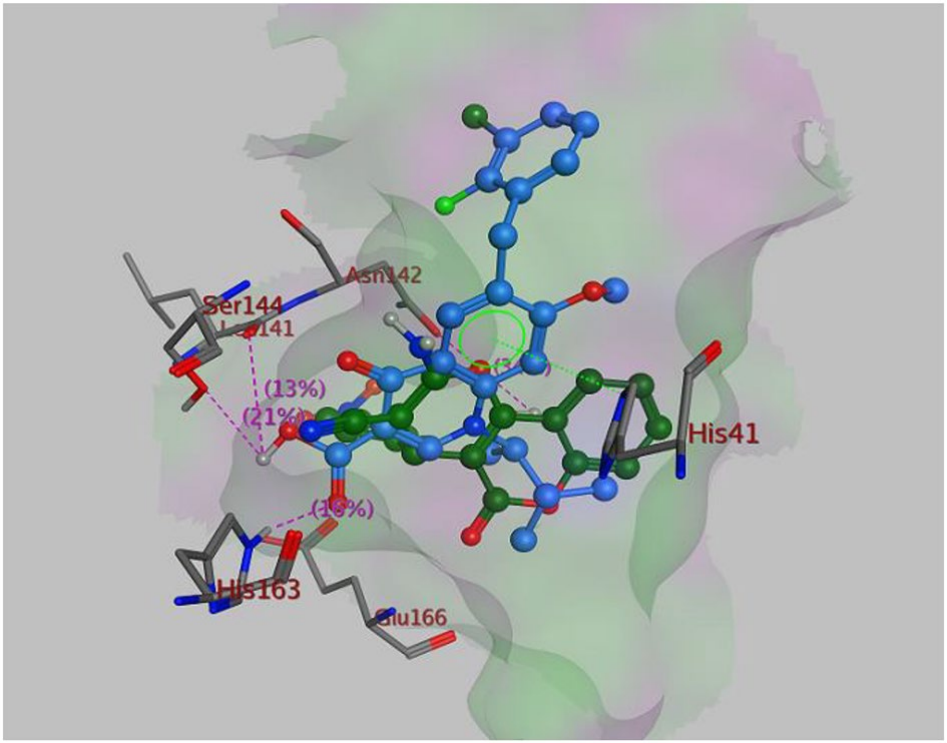
Superimposition of B_1_ (green) and elvitegravir (blue) within the enzyme binding site (PDB code: 5rgi).

Table 7 displays the docking scores of all compounds and A2, B1 and C4 was identified as the most probable COVID-19 inhibitor, with binding energies A2 (−8.1 and −7.4 kcal/mol), B1 (−8.2 and −8.1 kcal/mol) and C4 (−7.5 and −7.0 kcal/mol) in compare with remdesivir (−7.4 and −8.0 kcal/mol) and elvitegravir (−8.0 and −7.4 kcal/mol) for 5rgi and 6lze, respectively. Figure 16 illustrates the docked image of A2, B1 and C4 and the residues implicated in the interaction which includes π–π stacking interaction, cation–π interactions and hydrogen bond. Residues involved in these interactions are His163, Phe140, Glu166, His41, Ser144, Leu141 and Cys145 in the active site of 5rgi as seen in the 3D diagram. For comparison, the standard elvitegravir creates similar binding pose in the active site pocket forming hydrogen bonds and also cation–π interaction with Asn142, His163, Ser144, Leu141 and His41 of the target. Figure 17 illustrates the superimposition of compound B1, the most probable compound, and elvitegravir within the targeted enzyme binding site (PDB code: 5rgi).

**Table 7:** RMSD values and binding energies of synthesized compounds A_1–7_, B_1–7_ and C_1–7_ in compare with remdesivir and elvitegravir as the standard anti-covid-19 medicine.

PDB code		5rf3	5rgi	6lze	6m0k
RMSD		1.87	0.31	1.13	1.58
Binding energy (Kcal mol^−1^)	A_1_	−7.5	−7.9	−7.6	−7.1
	A_2_	−7.5	−8.1	−7.4	−7.4
	A_3_	−8.0	−7.5	−7.2	−7.2
	A_4_	−7.3	−6.8	−6.9	−6.5
	A_5_	−7.5	−7.1	−7.5	−6.6
	A_6_	−7.4	−7.1	−7.0	−6.6
	A_7_	−7.3	−7.1	−6.9	−6.6
	B_1_	−8.2	−8.2	−8.1	−7.8
	B_2_	−7.3	−7.9	−7.8	−7.4
	B_3_	−7.8	−8.1	−7.3	−7.2
	B_4_	−7.8	−7.6	−7.8	−7.9
	B_5_	−7.6	−7.9	−8.0	−8.0
	B_6_	−7.6	−8.3	−7.5	−7.6
	B_7_	−7.4	−8.0	−7.7	−7.4
	C_1_	−7.2	−6.4	−7.0	−6.9
	C_2_	−7.2	−6.4	−6.6	−6.5
	C_3_	−6.4	−6.0	−6.9	−6.0
	C_4_	−6.7	−7.5	−7.0	−6.6
	C_5_	−6.7	−6.1	−7.3	−6.3
	C_6_	−6.9	−6.4	−7.3	−6.2
	C_7_	−7.1	−6.7	−6.8	−6.5
	Elvitegravir	−7.3	−8.0	−7.4	−7.3
	Remdesivir	−7.2	−7.4	−8.0	−7.7

Based on the results of the docking study above, it was found that pyrano[3,2-*c*]chromene derivatives from all three categories demonstrated strong agreement in relation to both computed Gibbs binding energy and their ability to interact with the active site. These findings suggest that these compounds are capable of binding to the active site of 3CLpro/Mpro, as supported by our current molecular docking study results and warrant further in vitro and in vivo study against COVID-19.

## Conclusions

In this study, a novel acid–base catalyst with suitable potency was successfully synthesized. Remarkably, the binding of organic compounds to the surface of graphene oxide by covalent bonding was achieved in a safe and environmentally friendly manner without any harmful or hazardous substances. The newly prepared catalyst has shown impressive catalytic activity and proved to be very effective in three different reactions. The catalyst was thoroughly characterized and identified using various analytical techniques, including IR, SEM, XPS, XRD, EDS, CHN and Raman. In this study, the synthesis process of polyhydroquinolines, benzo[*b*]pyran and pyrano[3,2‑*c*] chromenes heterocyclic derivatives was investigated using GO -mor catalyst in an EtOH/H2O solvent. The use of GO -mor catalyst offered several advantages, such as high efficiency and easy product separation. The catalyst exhibited favorable acid–base properties, heterogeneity, and reusability. The simplicity and environmental friendliness of the catalyst synthesis also contribute to the attractiveness of this approach. Molecular docking analysis showed that the most likely compounds, pyrano[3,2-*c*]chromene derivatives, had significantly lower Gibbs binding energies among all three groups (Supplementary Information).

### Supplementary Information

Supplementary Information.

## Data Availability

All data generated or analyzed during this study are included in this published article.
