# Supplementary material for: Graphene oxide immobilized 2-morpholinoethanamine as a versatile acid–base catalyst for synthesis of some heterocyclic compounds and molecular docking study
Source: Sci Rep. 2023 Oct 20;13:17966. doi: 10.1038/s41598-023-44521-9 (PMC10589275; doi:10.1038/s41598-023-44521-9)
Supplement: Supplementary file 1 — Supplementary Information. [file 41598_2023_44521_MOESM1_ESM.docx]

**Supplementary Materials**

**Graphene Oxide Immobilized 2-Morpholinoethanamine as A Versatile Acid-Base Catalyst for Synthesis of Some Heterocyclic Compounds and Molecular Docking Study**

Leila Amiri-Zirtol^[[1]](#footnote-1)1^, Tahereh Solymani Ahooie^2^, Elham Riazimontazer^3^, Mohammad Ali Amrollahi^1^, Bibi-Fatemeh Mirjalili^1^

1- Department of Chemistry, Yazd University, Yazd, Iran

2-Chemistry and chemical Engineering Research Center of Iran

3-Biotechnology Research Center, Shiraz University of Medical Sciences, Shiraz, Iran, Department of Medicinal Chemistry, School of Pharmacy, Shiraz University of Medical Sciences, Shiraz, Iran


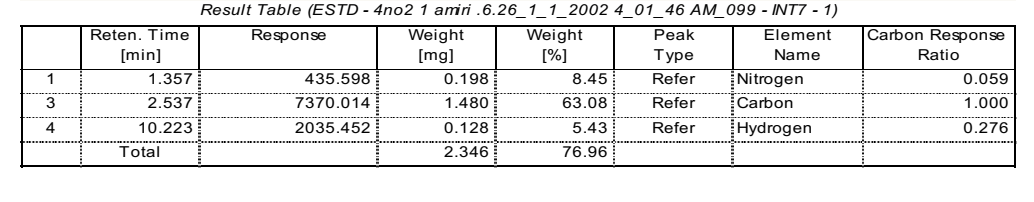


The CHN spectrum of 2-Amino-7,7-dimethyl-4-(4-nitrophenyl)-5-oxo-5,6,7,8-tetrahydro-4*H*-chromene-3-carbonitrile ***(1a)***

***
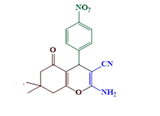
***
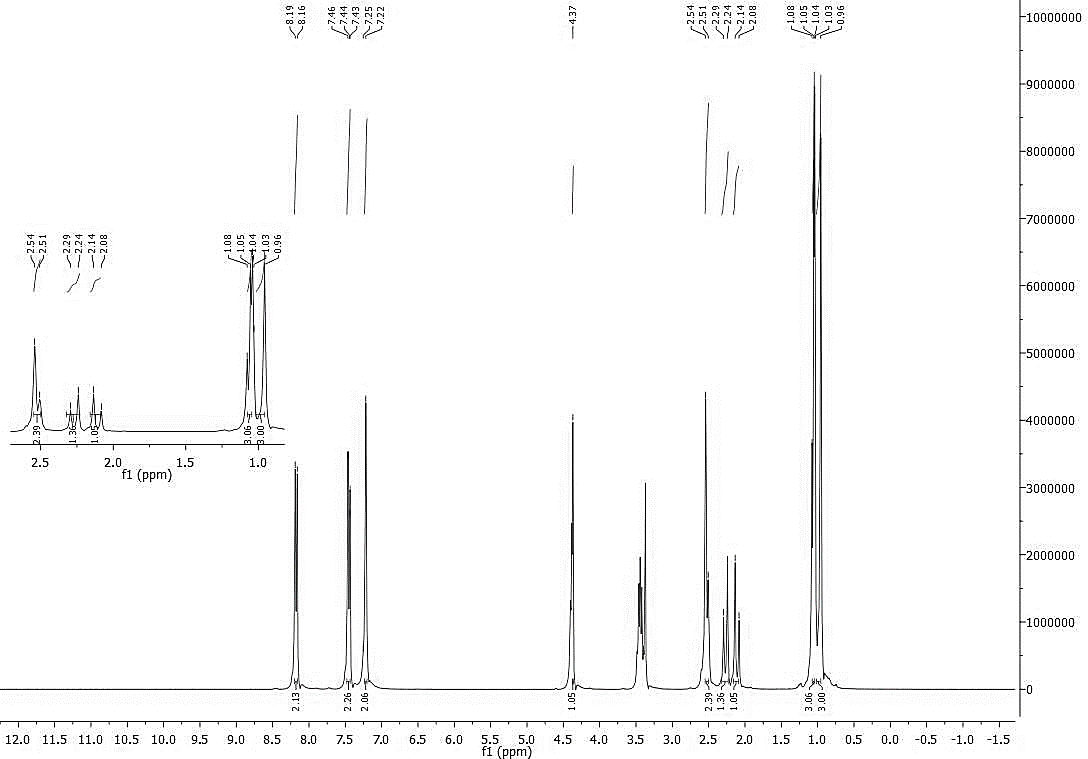


^1^H-NMR spectrum of 2-Amino-7,7-dimethyl-4-(4-nitrophenyl)-5-oxo-5,6,7,8-tetrahydro-4*H*-chromene-3-carbonitrile ***(1a)***


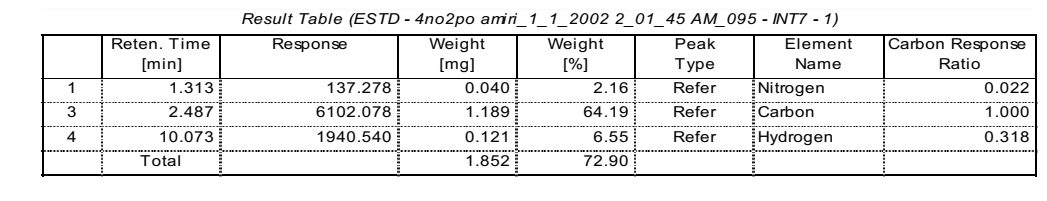


The CHN spectrum of Ethyl2,7,7‑trimethyl‑4‑(4‑nitrophenyl) -5‑oxo‑1,4,5,6,7,8‑hexahydroquinoline‑3‑carboxylate (1c)

**
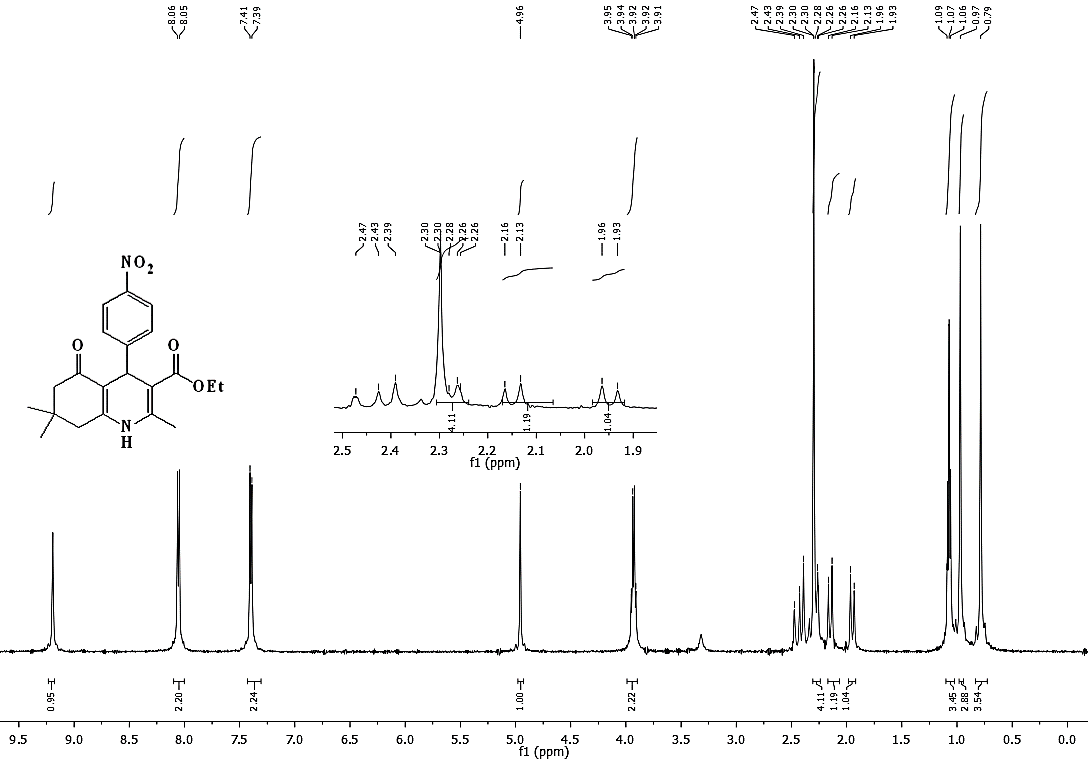
**

^1^H-NMR spectrum of Ethyl2,7,7‑trimethyl‑4‑(4‑nitrophenyl) -5‑oxo‑1,4,5,6,7,8‑hexahydroquinoline‑3‑carboxylate (1c)

**
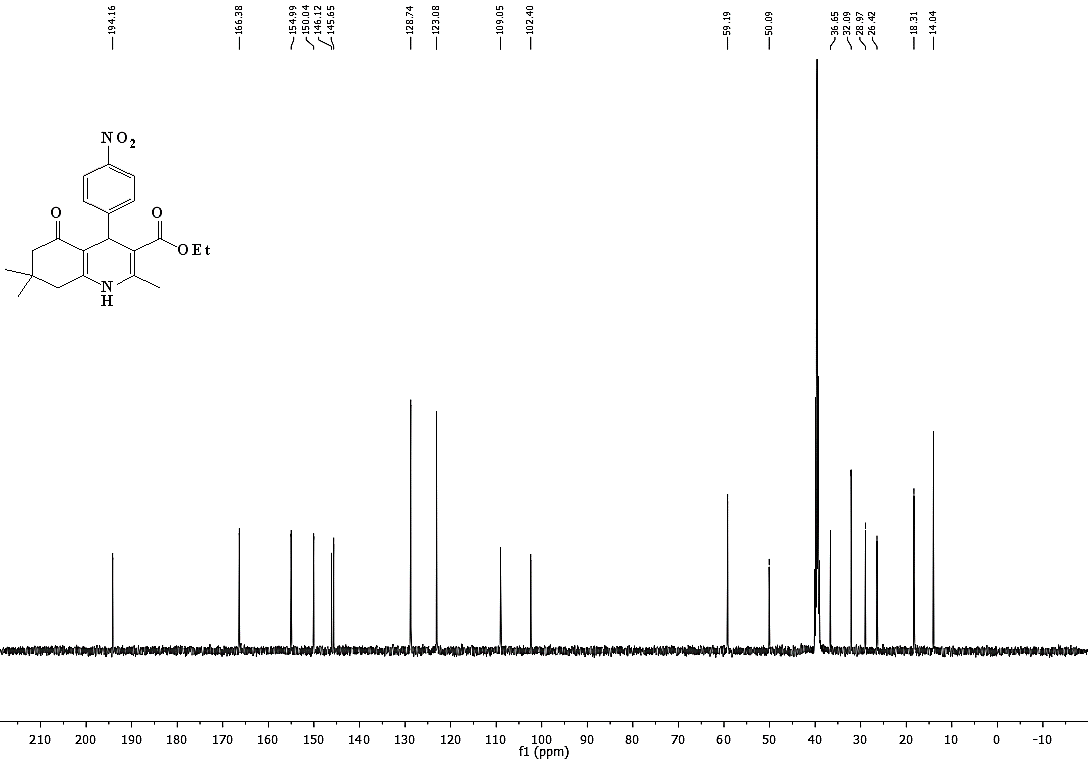
**

The ^13^C NMR spectrum of Ethyl2,7,7‑trimethyl‑4‑(4‑nitrophenyl) -5‑oxo‑1,4,5,6,7,8‑hexahydroquinoline‑3‑carboxylate (1c)


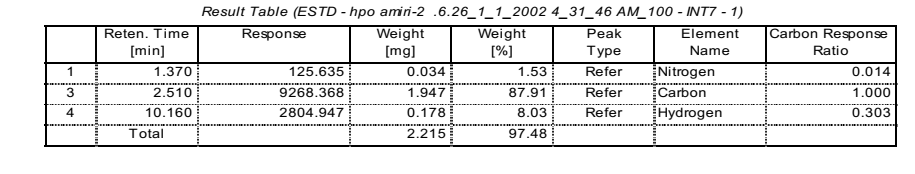


The CHN spectrum of Ethyl 2,7,7-trimethyl-5-oxo-4-phenyl-1,4,5,6,7,8-hexahydroquinoline -3-carboxylate (3c)

**
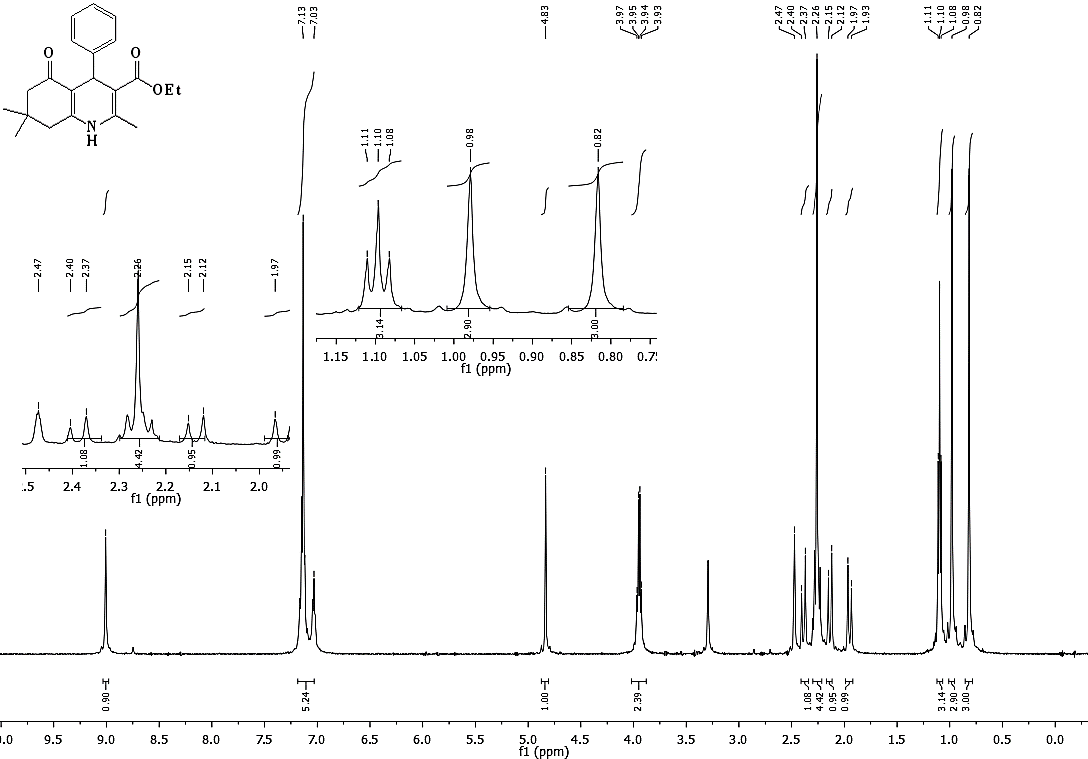
**

^1^H-NMR spectrum of Ethyl 2,7,7-trimethyl-5-oxo-4-phenyl-1,4,5,6,7,8-hexahydroquinoline -3-carboxylate (3c)

**
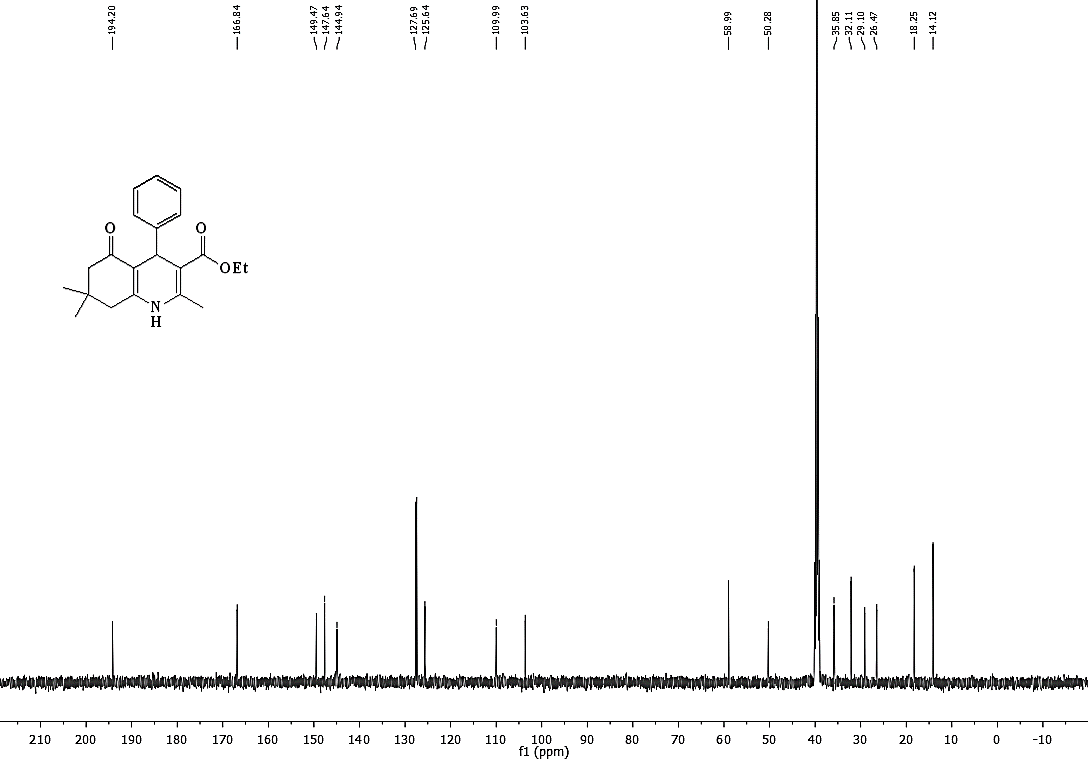
**

The ^13^C NMR spectrum of Ethyl 2,7,7-trimethyl-5-oxo-4-phenyl-1,4,5,6,7,8-hexahydroquinoline -3-carboxylate (3c)


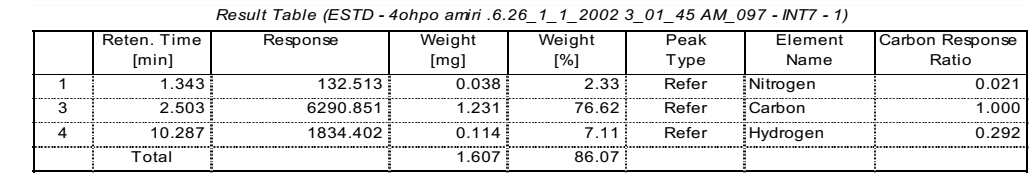


The CHN spectrum of Ethyl 4-(4-hydroxyphenyl)-2,7,7-trimethyl-5-oxo-1,4,5,6,7,8-hexahydroquinoline-3-
carboxylate (4c)

**
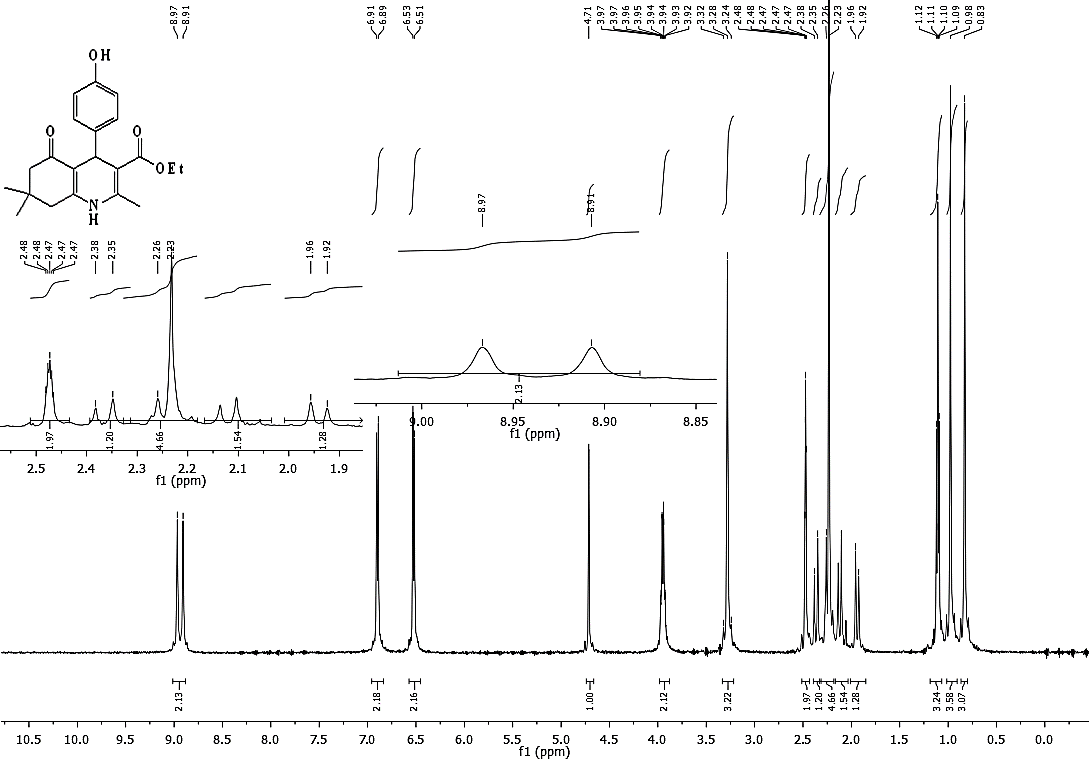
**

^1^H-NMR spectrum of Ethyl 4-(4-hydroxyphenyl)-2,7,7-trimethyl-5-oxo-1,4,5,6,7,8-hexahydroquinoline-3-
carboxylate (4c)

**
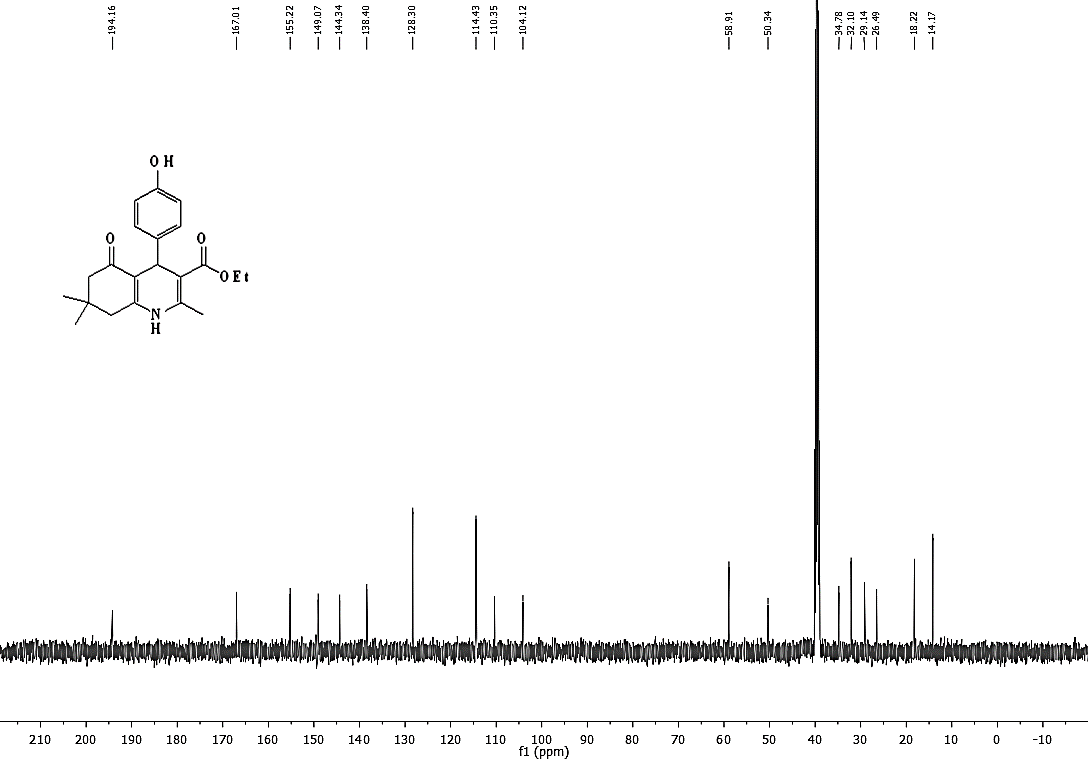
**

The ^13^C NMR spectrum of Ethyl 4-(4-hydroxyphenyl)-2,7,7-trimethyl-5-oxo-1,4,5,6,7,8-hexahydroquinoline-3-
carboxylate (4c)

1. Corresponding author.
   E-mail address: [l.amiri@stu.yazd.ac.ir](l.amiri@stu.yazd.ac.ir%20), [l.amiri@stu.yazd.ac.ir](mailto:l.amiri@stu.yazd.ac.ir) (L. Amiri-Zirtol) [↑](#footnote-ref-1)
